# First PCR-Confirmed Case of Feline Hemoplasmosis in Bosnia and Herzegovina with a Long-Term Follow-Up

**DOI:** 10.3390/vetsci13050410

**Published:** 2026-04-22

**Authors:** Dajna Preldžić, Zinka Maksimović, Maid Rifatbegović, Denis Čamo

**Affiliations:** 1 Department of Clinical Sciences of Veterinary Medicine, Veterinary Faculty, University of Sarajevo, 71 000 Sarajevo, Bosnia and Herzegovina; denis.camo@vfs.unsa.ba; 2 Department of Pathobiology and Epidemiology, Veterinary Faculty, University of Sarajevo, 71 000 Sarajevo, Bosnia and Herzegovina; zinka.maksimovic@vfs.unsa.ba (Z.M.); maid.rifatbegovic@vfs.unsa.ba (M.R.)

**Keywords:** Candidatus *Mycoplasma haematominutum*, PCR, FIV, enrofloxacin, non-regenerative anemia, feline hemoplasmosis, Bosnia and Herzegovina

## Abstract

Hemotropic mycoplasmas are Gram-negative bacteria and are recognized as primary causative agents of feline infectious anemia, with a worldwide distribution and variable pathogenicity. This case report describes an unusual clinical presentation of hemoplasmosis in a seventeen-year-old spayed female cat that presented with severe flea infestation accompanied by marked weakness and lethargy. It was febrile, tachycardic, and tachypnoic, with prominent pallor of the mucous membranes. Laboratory analysis revealed severe, non-regenerative, microcytic and (falsely) hyperchromic anemia, with mild lymphopenia and slightly increased plateletcrit. The cat also tested positive for feline immunodeficiency virus (FIV). Blood smear examination raised suspicion of hemoplasmosis, which was later confirmed and identified by polymerase chain reaction (PCR) as Candidatus *Mycoplasma haematominutum*. Following the treatment of ectoparasites, the cat was successfully treated with enrofloxacin and erythropoiesis-stimulating agents (ESAs), avoiding blood transfusion, and afterwards made a full clinical recovery. Although immunocompromised due to FIV, the cat lived for an additional five years without further relapses. To the best of the authors’ knowledge, this is the first documented and PCR-confirmed case of feline hemoplasmosis in Bosnia and Herzegovina. It also highlights the need for conducting a larger study in this region to evaluate hemoplasma prevalence in this species.

## Simple Summary

Feline patients may frequently develop anemia in association with a variety of underlying concurrent conditions, primarily as a direct consequence of metabolic disorders or infectious agents such as hemoplasmas. Additionally, anemia may arise as a part of a primary myeloproliferative disease, particularly associated with retroviral infections. Recent data from owners and veterinarians indicate that feline hemoplasmosis, despite its worldwide occurrence, is not routinely considered as the differential diagnosis for anemia in cats of Bosnia and Herzegovina. The infection is often underdiagnosed and overlooked, primarily due to the absence of reliable rapid testing methods and lack of hemoplasmosis PCR testing availability in Bosnia and Herzegovina, prior to this study. This is the first reported case of PCR-confirmed hemoplasmosis in Bosnia and Herzegovina. It further provides detailed information on patients’ signalment, clinical and molecular diagnostic approaches, treatment, and long-term follow-up. Despite being immunocompromised due to feline immunodeficiency virus infection, no recurrence of hemoplasmosis or retroviral reactivation was observed over an almost five-year period. This case also emphasizes the importance of considering hemoplasmosis in anemic cats, particularly those that do not respond to the treatment, and demonstrates the value of molecular methods in establishing definitive diagnosis.

## 1. Introduction

Hemotropic mycoplasmas are Gram-negative bacteria, also referred to as hemoplasmas, and are recognized as causative agents of feline infectious anemia. Due to their hemotropic nature, they parasitize the surface of red blood cells [1]. The three most common feline hemoplasmas identified to date are *Mycoplasma haemofelis (Mhf*), Candidatus *Mycoplasma haematominutum* (*C*Mhm), and Candidatus *Mycoplasma turicense* (*C*Mt) [1,2,3]. These organisms have been reported worldwide [1]. The highest prevalence of feline hemoplasmosis in Europe has been reported in Portugal (43.43% and 27.1%) [4,5]. The most commonly reported hemoplasma in European cats was *C*Mhm, with the lowest prevalence noted in Catalonia, Spain (6/135; 4.4%) [6] and the highest in Portugal (33/320; 41.56%) [4]. Among the other European countries following the same trend of CMhm being the most frequently identified hemoplasma are the Czech Republic (*C*Mhm 35.6%) [7], Turkey (*C*Mhm 17.7%) [8], Latvia (*C*Mhm 15%) [9], Northern Serbia (*C*Mhm 12.6%) [10], and Greece (*C*Mhm 10.3%) [11]. Although *Mhf*, CMhm, and CMt are widespread among wild cats in Europe [12], none of these hemoplasmas were identified in European wild cats in Bosnia and Herzegovina [13]. Phylogenetic analyses revealed that those identified in four individuals instead represent two different nucleotide sequences: type A forms a separate clade genetically distinct from the *haematominutum* group (Candidatus *Mycoplasma haematoparvum* and Candidatus *Mycoplasma haematominutum*) and, in contrast, type B belongs to the *haemofelis* group (uncultured *Mycoplasma* sp. from raccoons and feline hemoplasmas, such as Candidatus *Mycoplasma turicensis*). These hemoplasmas may represent previously undescribed and novel species [13]. Transmission occurs primarily via horizontal routes [1,2], although vertical transmission has also been documented [14]. Arthropods, particularly fleas (*Ctenocephalides felis*), are implicated as major vectors [15]. Iatrogenic transmission through blood transfusion has also been reported [3]. Older cats are considered as a target group due to their weaker immune response. However, infections in these cats are often subclinical, with *C*Mhm being the most commonly identified hemoplasma. In contrast, younger cats tend to show more overt clinical signs due to their naïve immune system and susceptibility to the more pathogenic *Mhf* [16]. Male cats are more predisposed to infection due to territorial aggression, with saliva identified as a potential source of transmission [17]. Consequently, outdoor lifestyle and the aforementioned pathways are considered risk factors for hemoplasma infection [1,2,3]. *Mhf* is the most pathogenic hemoplasma, capable of inducing fatal hemolytic anemia in acutely infected cats, even in the absence of concurrent disease [1]. Chronic infection is typically mild, particularly during the carrier phase, and may occur without clinically evident anemia [1]. Candidatus species are generally less pathogenic [1,18], but can cause clinical disease in association with other infectious agents or retroviral immunodeficiency, such as feline immunodeficiency virus (FIV) [19,20,21], and less frequently Feline Leukemia Virus (FeLV) [2]. *C*Mhm infection concurrent with FeLV has been linked to myeloproliferative disorders [22]. Moreover, *C*Mhm alone has been shown to cause anemia [23]. Clinical manifestations of hemoplasmosis include moderate to severe hemolytic anemia, often accompanied by pallor of the mucous membranes [1]. Hemolysis may occur extravascularly [24] or intravascularly [25]. Despite the hemolytic nature of the anemia, icterus is rarely observed [1,2]. Severe cases may present with tachycardia, tachypnea, anorexia, dehydration, and intermittent pyrexia. Clinical signs, combined with severe anemia, risk factors, and medical history, often place hemoplasmosis high on the differential diagnosis list [1,2]. Definitive diagnosis requires PCR testing, as blood smear examination is considered insensitive due to fluctuations in bacteremia and its inability to reliably differentiate hemoplasmas [1]. This case report describes the first clinically evident hemoplasma infection confirmed by PCR in a cat in Bosnia and Herzegovina, with complete recovery.

## 2. Case Description

**Figure 1:**
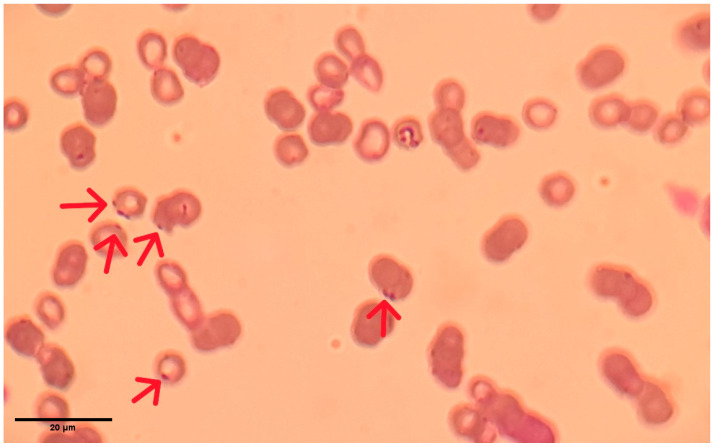
*Haemoplasma* spp. observed in a stained peripheral blood smear from a domestic cat. The organisms appear as small, dark blue structures adherent to the surface of erythrocytes (red arrows).

A 17-year-old, domestic shorthaired, spayed female cat was referred to the Clinic for Internal Diseases, Oncology, and Emergency Medicine of Small Animals, Veterinary Faculty, University of Sarajevo, for abdominal ultrasonography due to severe lethargy and anorexia. The interval from onset to first visit was two days, following referral by the primary veterinarian. At presentation, the cat was recumbent, very weak, lethargic, and depressed. Consequently, the owner consented to a complete clinical examination, diagnostics, and treatment. The cat had been kept strictly indoors for the past three years, following 14 years of outdoor access. It was the only cat in the household. On physical examination, severe flea infestation was noted. The mucous membranes were markedly pale, rendering capillary refill time immeasurable. The cat was febrile (39.7 °C), dehydrated (5%), and tachycardic with a weak pulse, and it had a mildly increased respiratory rate. The body condition score was 3/9, with moderate sarcopenia. Thoracic auscultation revealed normal rhythm with accentuated cardiac sounds and vesicular breath sounds. Abdominal palpation was non-painful and revealed no abnormalities. A complete blood count (Idexx ProCyte Dx^®^, IDEXX Laboratories Inc., Westbrook, ME, USA) demonstrated severe non-regenerative anemia (hematocrit 6%; reference range 30.3–52.3%), appearing falsely hyperchromic due to pronounced microcytosis. Mild lymphopenia was present, and plateletcrit (PCT%) was slightly elevated (Table 1). Serological testing using the SNAP FIV/FeLV Combo Test (IDEXX Laboratories Inc.) revealed that the cat tested positive for feline immunodeficiency virus (FIV) and negative for Feline Leukemia Virus (FeLV). Serum biochemistry (Catalyst One Chemistry Analyzer, IDEXX Laboratories Inc.) was within reference ranges. Abdominal ultrasonography showed no abnormalities. Microscopic examination of a Diff-Quik-stained blood smear revealed reduced erythrocyte number, microcytosis, and numerous dark blue microorganisms adherent to erythrocyte membranes (Figure 1). Based on clinical and laboratory findings, hemoplasmosis was suspected. The blood sample was submitted for PCR analysis on the same day, prior to initiation of treatment. Antimicrobial therapy (enrofloxacin) was started only after sample submission, once confirmation was obtained that the laboratory had received the sample, thereby avoiding any potential interference with PCR results. Results of PCR testing were available within seven days. DNA was extracted from EDTA-anticoagulated blood using the DNeasy Blood and Tissue Kit (QIAGEN GmbH, Hilden, Germany). Universal hemoplasma screening was conducted via SYBR Green real-time PCR [26] using the QuantiNova SYBR Green PCR Master Mix (QIAGEN GmbH, Hilden, Germany) and primers (Eurofins, MWG, Operon, Louisville, KY, USA). Identification of feline hemoplasmas, *Mhf*, *C*Mhm, and *C*Mt [5], was performed by specific real-time PCR assays, using Luna^®^ Universal Probe qPCR Master Mix (New England Biolabs, Ipswich, MA, USA), primers, and probes specific to *Mhf*, *C*Mhm, and *C*Mt (Eurofins, MWG, Operon, Louisville, KY, USA). A sample was considered positive for *Haemoplasma* spp., when the cycle threshold (Ct) value was ≤35, whereas positivity for *C*Mhm was defined at Ct ≤ 40. The active infection was confirmed, and *C*Mhm was identified. Supportive therapy was initiated due to the patient’s weakness and anorexia. Flea infestation was treated with topical moxidectin and imidacloprid (Advocate™, Elanco, Indianapolis, IN, USA). Intravenous fluid therapy with Ringer’s lactate (B. Braun Melsungen AG, Melsungen, Germany) was administered according to protocols for severely anemic patients [19], maintaining hydration and microvascular perfusion, without exacerbating anemia [27]. Darbepoetin alfa (Aranesp^®^, 10 µg/0.4 mL; Amgen Europe B.V., Netherlands) was administered subcutaneously at 1 µg/kg once weekly (for two weeks) until hematocrit exceeded 20%, which was discontinued on the thirteenth day of treatment, when control hematology analysis was performed. Darbepoetin alfa was accompanied by intramuscular hydroxocobalamin (OHB_12_ injectable solution 2500 µg/2 mL, Galenika a.d., Belgrade, Serbia) supplementation (250 µg once weekly) for the full course of the treatment. Enrofloxacin (Enroxil Flavor 15 mg, KRKA, Novo Mesto, Slovenia) was given orally at 5 mg/kg q24h for 28 days. Due to financial constraints, blood transfusion was not performed initially, though the owner was advised it would be necessary if clinical deterioration occurred. Marked clinical improvement was observed within the first week of treatment, including increased appetite, responsiveness, and activity. Mucous membranes were no longer severely pale. Hematological improvement (hematocrit increase from 6% to 25.9%) was documented on day 13 of treatment (Table 1) and revealed mild normocytic anemia, without additional abnormalities. The cat remained clinically healthy for five years without recurrence of hemoplasmosis.

## 3. Discussion

Feline hemoplasmas identified to date have variable pathogenicity. *Mhf* is generally considered the most pathogenic, capable of inducing severe, potentially fatal anemia in acutely infected cats, although clinical signs may be absent in chronic infections or immunocompetent individuals [1,2,3]. In contrast, *C*Mhm, despite its high worldwide prevalence, is less pathogenic, with infections often remaining subclinical. Similarly, monoinfections with *C*Mt, the least prevalent feline hemoplasma, are typically subclinical [1,2,3]. Concurrent illnesses or immunosuppressive conditions, such as retroviral infections or hemoplasma coinfections, can exacerbate disease severity and lead to overt clinical signs [21,22]. Our patient was immunocompromised due to FIV infection and coinfected with hemoplasma, which likely contributed to the complex clinical presentation. Severe flea infestation represented an additional risk factor, as arthropod vectors play the main role in the horizontal transmission of feline hemoplasmas and can exacerbate anemia as well [15,25]. The cat’s history of outdoor access for 14 years further increased the likelihood of exposure to infectious agents and aggressive encounters with infected cats, predisposing her to both FIV and hemoplasma infection [15,28]. Hematological findings in this case differed slightly from those reported in previous studies [29,30]. Pathogenic hemoplasma infections typically result in regenerative, hypochromic, macrocytic anemia, which may be fatal [1]. In contrast, our patient exhibited severe (hematocrit 6%) non-regenerative, microcytic, and falsely hyperchromic anemia, indicating consideration of emergent transfusion. Severe cases of non-regenerative anemia have been reported in hemoplasma monoinfections and in retroviral coinfections [2,4,31,32]. In this case, anemia may have been pre-regenerative, secondary to infection, or truly non-regenerative due to FIV-associated myeloproliferative disease. Hematocrit normalized by the end of treatment, although anemia remained non-regenerative during therapy. The apparent hyperchromia was likely artifactual, resulting from microcytosis and analyzer overestimation of hemoglobin concentration [33]. However, microcytosis could be the result of chronic blood loss, due to flea infestation, decreased erythropoiesis observed in certain inflammatory stages, or reduced red blood cell (RBC) lifespan, which is most likely due to excessive activation of the reticuloendothelial system (RES), resulting in premature removal of RBCs from the circulation [32]. Consequently, darbepoetin alfa was chosen because reduced endogenous erythropoietin production was suspected to be associated with coinfection and chronic inflammation, owing to its longer half-life compared with epoetin [32]. No side effects were observed after administration. Leukopenia, including lymphopenia and basopenia, is frequently observed in hemoplasmosis, while monocytosis may also occur [1]. Mild lymphopenia was evident in this case, with no other leukocyte abnormalities. No abnormalities were detected in biochemistry results. Cytology is generally considered insensitive for hemoplasma diagnosis. *Mhf* is most frequently identified on blood smears, followed by *C*Mhm because of the rapid fluctuation in bacteriemia, while *C*Mt is rarely detected due to low bacterial load [1,2]. In this case, microscopic examination revealed dark blue epierythrocytic microorganisms suggestive of *Mhf*, consistent with previous reports [1,2]. However, PCR confirmed *C*Mhm infection. This discrepancy may reflect early smear preparation, interpretation bias, or exacerbated clinical signs due to FIV coinfection. Antibiotic therapy for feline hemoplasmosis typically involves doxycycline or fluoroquinolones, with prolonged courses (≥28 days) recommended to maximize clinical resolution and bacteremia clearance. However, not all hemoplasmas respond equally to these antibiotics. Doxycycline is most effective against *Mhf* and *C*Mt [1,2,3], while fluoroquinolones (pradofloxacin, marbofloxacin) are preferred for *C*Mhm [1]. Enrofloxacin, although effective against *Mhf*, is less commonly recommended due to rare reports of retinal toxicity and acute blindness [1,34]. Combination therapy may be considered if monotherapy fails [1,2]. In Bosnia and Herzegovina, veterinary doxycycline was unavailable, and human formulations posed dosing challenges. Marbofloxacin and pradofloxacin were not yet accessible; thus, enrofloxacin was administered. No ophthalmological adverse effects were observed during or after treatment. The cat showed clinical improvement, with hematological parameters returning to reference ranges by the end of therapy, suggesting enrofloxacin efficacy in *C*Mhm infection. Although follow-up PCR testing was not performed to confirm bacteriological clearance, the patient achieved sustained long-term clinical remission. The favorable treatment response supports the hypothesis that concurrent FIV infection enhanced hemoplasma pathogenicity, facilitating clinical disease expression. Despite the absence of FIV viral load assessment, regular annual examinations revealed no evidence of disease progression, with the cat remaining clinically stable and laboratory findings in reference range. The patient remained free of hemoplasmosis relapses and FIV flare-ups for five years following treatment.

## 4. Conclusions

This case report represents the first documented and PCR-confirmed occurrence of hemoplasmas in the feline population of Bosnia and Herzegovina. Despite being considered less pathogenic, it has been reported that Candidatus *Mycoplasma haematominutum* may induce anemia as a sole agent; therefore, hemoplasmas should be considered as a potential cause of anemia even in the absence of concurrent diseases. The presence of hemoplasmas in cats of this region underscores the need for conducting larger epidemiological studies, as these microorganisms may persist in the carrier state in asymptomatic cats, consequently causing clinically apparent disease primarily with concurrent conditions including retroviral infections in immunocompromised animals or neoplastic diseases. The absence of follow-up PCR testing to confirm complete bacteriological clearance poses a limitation of this study. Although clinical improvement was observed and laboratory findings remained unremarkable thereafter, complete elimination of the pathogen could not be definitively verified. Future studies incorporating serial PCR testing would be valuable to better assess treatment efficacy and long-term infection dynamics. The uncontrolled growth of stray and feral cat population in this region, along with inadequate control of ectoparasite infestation and other infectious diseases, as well as low neutering/spaying rates, creates favorable conditions for vector-borne pathogens to thrive. Given their clinical significance and impact on feline health, hemoplasmas should be routinely incorporated in infectious disease screening protocols, alongside effective quarantine, anti-ectoparasitic and treatment strategies to prevent further transmission.

## Figures and Tables

**Table 1:** Hematological analysis before and during enrofloxacin hemoplasmosis treatment.

Parameters	Reference Interval	Pre-Treatment Values	Values 13 Days of Treatment
RBC	6.54–12.20 × 10^12^/L	2.58	5.47
Hematocrit	0.303–0.523 L/L	0.067	0.259
Hemoglobin	98–162 g/L	31	72
MCV	35.9–53.1 fL	26.0	47.3
MCH	11.8–17.3 pg	12.0	13.2
MCHC	281–358 g/L	463	278
RDW	15.0–27.0%	16.2	36.1
Reticulocytes	3.0–50.0 K/μL	16.3	12.6
Reticulocyte-hemoglobin	13.2–20.8 pg	14.0	14.7
WBC	2.87–17.02 × 10^9^/L	8.03	7.24
Neutrophils	2.30–10.29 × 10^9^/L	6.20	4.77
Lymphocytes	0.92–6.88 × 10^9^/L	0.91	1.15
Monocytes	0.05–0.67 × 10^9^/L	0.49	0.19
Eosinophils	0.17–1.57 × 10^9^/L	0.37	1.06
Basophils	0.01–0.26 × 10^9^/L	0.06	0.07
Platelets	151–600 × 10^9^/L	507	358
MPV	11.4–21.6 fL	19.1	16.5
Plateletcrit	0.17–0.86%	0.97	0.59

MCHC = mean corpuscular hemoglobin concentration; MCH = mean corpuscular hemoglobin; MCV = mean corpuscular volume; MPV = mean platelet volume; RBC = red blood cell; RDW = red cell distribution width; WBC = white blood cell.

## Data Availability

The raw data supporting the conclusions of this article will be made available by the authors on request.

## Abbreviations

The following abbreviations are used in this manuscript:

*Mhf*	*Mycoplasma haemofelis*
*C*Mhm	Candidatus *Mycoplasma haematominutum*
*C*Mt	Candidatus *Mycoplasma turicensis*
FIV	Feline immunodeficiency virus
FeLV	Feline Leukemia Virus
PCR	Polymerase chain reaction
DNA	Desoxyribonucleic acid
EDTA	Ethylenediaminetetraacetic acid
